# Nested ensemble selection: An effective hybrid feature selection method

**DOI:** 10.1016/j.heliyon.2023.e19686

**Published:** 2023-09-09

**Authors:** Firuz Kamalov, Hana Sulieman, Sherif Moussa, Jorge Avante Reyes, Murodbek Safaraliev

**Affiliations:** a Department of Electrical Engineering, Canadian University Dubai, Dubai, United Arab Emirates; b Department of Mathematics and Statistics, American University of Sharjah, Sharjah, United Arab Emirates; c Department of Automated Electrical Systems, Ural Federal University, Yekaterinburg, Russian Federation

**Keywords:** Feature selection, Ensemble selection, Random forest, Synthetic data, Machine learning, Filter method, Wrapper method

## Abstract

It has been shown that while feature selection algorithms are able to distinguish between relevant and irrelevant features, they fail to differentiate between relevant and redundant and correlated features. To address this issue, we propose a highly effective approach, called Nested Ensemble Selection (NES), that is based on a combination of filter and wrapper methods. The proposed feature selection algorithm differs from the existing filter-wrapper hybrid methods in its simplicity and efficiency as well as precision. The new algorithm is able to separate the relevant variables from the irrelevant as well as the redundant and correlated features. Furthermore, we provide a robust heuristic for identifying the optimal number of selected features which remains one of the greatest challenges in feature selection. Numerical experiments on synthetic and real-life data demonstrate the effectiveness of the proposed method. The NES algorithm achieves perfect precision on the synthetic data and near optimal accuracy on the real-life data. The proposed method is compared against several popular algorithms including mRMR, Boruta, genetic, recursive feature elimination, Lasso, and Elastic Net. The results show that NES significantly outperforms the benchmarks algorithms especially on multi-class datasets.

## 1 Introduction

The aim of feature selection is to reduce the number of features under consideration. It can lead to better interpretability of the model, lower computational load, lower chance of overfitting, and enhanced model accuracy. Ideally, all possible feature subsets should be considered before choosing the best one. However, given *n* features there are $2^{n}$ possible subsets which becomes an intractable problem even for moderate values of *n*. Thus, there exist many heuristics that attempt to find the optimal subset without going through the exhaustive search. While some feature selection algorithms have shown good results, there remains a significant room for improvement.

It has been shown that feature selection algorithms can often differentiate between the relevant and irrelevant features. On the other hand, the algorithms fail to distinguish the relevant variables from the redundant and correlated variables [27]. To address this issue, we propose a two-step approach that combines filter and wrapper methods to achieve high precision with relatively low computational complexity. The proposed method, called Nested Ensemble Selection (NES), can effectively separate the relevant features from all the rest including irrelevant, redundant, and correlated features. The results of numerical experiments show that the proposed algorithm correctly identifies all the relevant features in synthetic datasets. In addition, the algorithm achieves near optimal results on real-life data.

There exists a trade-off between the precision and speed of feature selection algorithms. While enlarging the search space of feature subsets increases the likelihood of finding the optimal subset, it requires longer computing times. To overcome this challenge, we leverage a key observation that it is possible to quickly separate the relevant features from the irrelevant ones using a filter approach. Filter approaches operate on small search spaces thereby providing a fast method for evaluating feature importances. However, filter methods are not effective at distinguishing between the relevant and redundant and correlated features. Therefore, in the second stage of our algorithm, we employ a wrapper method that searches through the space of all subsets of a fixed size. Since the wrapper method is applied on the filtered features the computing times are exponentially reduced compared to the original set of features. As a result, we obtain an algorithm that performs a detailed search but on a reduced subset of features.

One of the key issues in feature selection is determining the number of features to be selected. Although there exist several techniques that attempt to help choose the optimal number of features, it remains a largely open, and perhaps unanswerable, question [39]. Nevertheless, at least in the case of the datasets considered in our study, the proposed approach provides a robust heuristic for identifying the optimal number of features. In particular, we are able to definitively determine the optimal subset size by studying the plot of the out-of-bag accuracy. The key advantages of the proposed method are summarized below:

1. Achieves perfect precision in identifying the relevant features (Table 9).
2. Provides an effective mechanism for identifying the number of relevant features.
3. Applies to both binary and multi-class datasets.

The paper is structured as follows. In Section 2, we present a brief overview of the current literature related to feature selection. In Section 3, we provide the details of the proposed feature selection algorithm. In Section 4, we present the results of numerical experiments evaluating the performance of the proposed algorithm. Section 5 concludes the paper.

## 2 Literature

Feature selection is key component in data science and machine learning applications in multiple fields including gene expression [4], intrusion detection [25], internet of things [35], and others. Given its importance, there exist many algorithms for feature selection in the literature. The existing approaches can be grouped into three major categories: filter, wrapper, and embedded methods. Filter methods use a univariate metric such as mutual information [41] or $\chi^{2}$ [26], while wrapper methods use a classifier to evaluate individual features. Embedded methods such as lasso perform automatic feature selection as part of the learning process.

A filter method based on neighborhood multi-granulation rough sets is proposed in [52] that uses a novel self-information measure for initial preprocessing followed by Fisher score to delete uncorrelated features. Another filter method called the Highest Wins was proposed in [33] for intrusion detection. The similarity between the expected and observed probabilities is quantified to generate feature scores in [47] which are used to evaluate feature importance. Normalized cross-covariance operator is used in [51] to measure nonlinear dependency between the dependent and independent variables. The maximum-relevance and minimum redundancy algorithm is an extension of the filter method which aims to maximize the mutual information a between feature subset and the dependent variable while minimizing the within-subset mutual information [37]. It has become one of the popular approaches in feature selection used both in academia [49] and industry [54]. The abundance of feature selection algorithms creates an issue for choosing the best approach. In [10], the authors compare 14 filter algorithms using 11 survival datasets and find that a simple variance based algorithm outperforms the more sophisticated techniques.

Support vector machines (SVM) are one of the popular base models for wrapper methods [6], [19]. An alternative wrapper method based on XGBoost classifier was proposed in [5]. Similarly, the authors in [46] combine XGBoost together with random forest and SVM to develop a wrapper method that is successfully applied to meteorological data.

Recently it has become increasingly popular to use nature inspired optimization techniques such as black widow optimization [21], seagull optimization [14], dispersed foraging swarm optimization [22], and others to feature selection. Another popular tactic has been to combine several approaches into a single hybrid method [1]. A number of methods have been proposed that combine individual filter scores into a vector as a single meta-score [2], [24]. The magnitude of the vector is used as a feature importance. In [15], the authors combine the ReliefF algorithm together with Principal Component Analysis to reduce dimensionality before applying the bagging classifier in network traffic data. The results show that feature selection can improve classification accuracy. A graph-theoretic method based on a two-step procedure that combines filter and wrapper methods was proposed in [13] to classify micro-array data. Beyond single-label learning feature selection has also been applied in other contexts such as multi-label, multi-view, unsupervised, and label distribution learning [31], [40], [53], [55].

The proposed feature selection algorithm differs from the existing filter-wrapper hybrid methods in its simplicity and effectiveness. The existing hybrid methods can be divided into 3 main categories: genetic, filter-driven, and fuzzy-based approaches. Genetic feature selection algorithms employ a heuristic to simultaneously optimize both filter and wrapper fitness functions [9], [18], [38]. Recent studies have tried to take this approach further by combining several genetic algorithms to search for the optimal feature subset [50]. While genetic algorithms can be appropriate in some cases, the added complexity of the algorithms does not justify the incremental improvements. In filter driven hybrid methods, innovative filter algorithms are utilized. The proposed filter algorithms employ new fitness functions to achieve more accurate selection [53]. Unfortunately, little theoretical justification is provided for the new fitness functions which raises the issue of reliability. Fuzzy-based hybrid algorithms attempt to apply the concepts of fuzzy logic to feature selection [3], [48]. As with the previous approaches, the added complexity of the fuzzy-based methods does not justify the incremental improvements in performance.

## 3 Nested ensemble selection

The proposed algorithm is based on the key observation that it is often possible that a feature selection algorithm efficiently discards the irrelevant features using a filter method. Since the filter method is computationally fast, it allows to reduce the feature space in a short amount of time. Afterwards, it remains to discard the redundant and correlated features which are left undetected by the filter method. Thus, the proposed method consists of two main stages:

1. Apply ensemble-based filter method (Equation (1)) to obtain individual feature scores. Select the top 20 features based on the scores and thereby discard the majority of the irrelevant features.
2. Apply random forest-based backward sequential search method on the top 20 features until the stopping criterion is achieved to discard the remaining redundant and correlated features.

### 3.1 Ensemble tree models

Tree-based ensemble classifiers play a crucial role in the proposed feature selection algorithm. In particular, random forest and extra tree classifiers are used to evaluate individual feature importances during the filtering stage. Random forest is an ensemble classifier that consists of a collection of trees. Each tree is fitted on a bootstrap sample of the data. The final model is constructed by aggregating the predictions of all the base trees [11]. Extra-trees classifier differs from random forest in the way each base tree is built. When searching for the best split to separate the samples of a node, random splits are drawn for each randomly selected feature and the best split among those is chosen [16]. As with most ensemble methods, the goal is to reduce overfitting. The parameter settings of the classifiers used in our study are provided in Table 1.

**Table 1:** The hyperparameter settings of the ensemble classifiers.

Parameter	Random Forest	Extra-trees
Number of base trees	100	100
Max depth	2	None
Bootstrap	Yes	Yes
Split criterion	Gini	Gini

### 3.2 Feature importance

Tree-based classifiers allow us to measure the reduction in impurity from splitting the data on a feature. We employ Gini index to calculate the impurity given by the following equation

$$G=\sum_{k=1}^{K}{\overset{ˆ}{p}}_{mk}(1-{\overset{ˆ}{p}}_{mk}),$$

where ${\overset{ˆ}{p}}_{mk}$ represents the proportion of training observations in the *m*th region that are from the *k*th class. Then feature importance is calculated as the total decrease in node impurity that results from splits over that variable, averaged over all trees in the ensemble [23]. The final feature score is obtained by taking the average of feature importances from random tree and extra tree classifiers:

(1) $$s(f_{k})=\frac{1}{2}(r(f_{k})+e(f_{k})),$$

where $s(f_{k})$ is final the score of the *k*th feature, $r(f_{k})$ and $e(f_{k})$ is the reduction in node impurity based on random forest and extra-trees classifiers, respectively.

### 3.3 The algorithm

**Figure 1:**
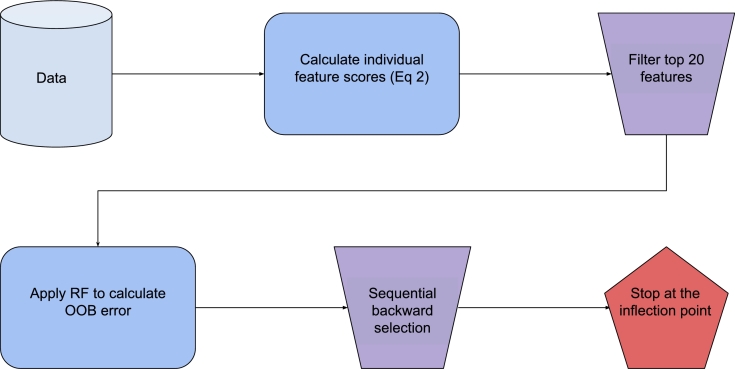
The Nested Ensemble Selection algorithm. First, the top 20 features are filtered using Equation (1). Then, sequential backward selection is applied based on RF until the stopping criterion.

The proposed feature selection algorithm, called Nested Ensemble Selection (NES), is illustrated in Fig. 1. It consists of two main stages. In the first stage, we calculate feature importances using random forest and extra-trees classifiers as discussed in Section 3.2. The individual feature scores obtained in the first stage are used to filter the top 20 features to be used for further analysis in the next stage. It has been shown that the top features selected via individual feature scoring are likely to contain the relevant features. In addition to the relevant variables, the top 20 features are also likely to contain the redundant, correlated, and a few irrelevant features. Thus, the goal of the second stage of feature selection is to separate the relevant variables from the rest of the top 20 features.

In the second stage, we perform a more detailed search among the top 20 features that are selected in the first stage. In particular, we perform a backward sequential feature selection. At each iteration, the least important feature is removed from the feature subset. The least important feature is determined based on the out-of-bag accuracy of random tree classifier. Concretely, for a given set of features of size *n*, we calculate the out-of-bag accuracy of a random forest classifier for all possible subsets of size n−1. The subset with the highest accuracy is chosen for the next iteration of the sequential search. The process is continued until only two features remain.

The heuristic for identifying the optimal size of the feature subset is based on the observation that there must be a significant reduction in classification accuracy whenever a relevant feature is removed from the dataset. Conversely, the classification accuracy should change little when an irrelevant or a redundant feature is removed from the subset. The use of backward elimination process provides an added robustness to the proposed approach as it is less likely to miss a relevant feature. Thus, the stopping criterion for the recursive feature elimination is given by a significant drop in the out-of-bag accuracy of a random forest classifier. We employ a visual approach to identify the optimal size of the feature subset. To this end, we consider the graph of the out-of-bag accuracy over the subset size obtained during the sequential search. The point of the sharpest decline in accuracy is considered as the size of the optimal subset. As demonstrated in the numerical experiments, the proposed approach produces consistent results with respect to different types of data. In each of the considered datasets, the graph of the out-of-bag accuracy shows a clear decline which indicates the stoppage of the backward elimination.

## 4 Numerical experiments

In this section, we test the performance of the proposed feature selection algorithm on several synthetic datasets with known relevant features and a real-life dataset. The results show that the NES algorithm correctly identifies all the relevant features in synthetic data. It also selects the top features in the real-life data that produce the same accuracy as the full set of features.

### 4.1 Data

We employ 4 synthetic datasets: ORAND, ANDOR, ADDER, and LED-16 that are described in [27]. Each dataset consists of 100 features and contains different combinations of relevant, redundant, correlated, and irrelevant features. The use of synthetic data allows us to judge exactly the correctness of the selected features. In addition, we employ an intrusion detection dataset (KDD 99) based on a simulated military network environment [43]. The details of the datasets are presented in Table 2.

**Table 2:** The details of the data used in the empirical testing.

Dataset	Relevant	Redundant	Correlated	Irrelevant	Total	Samples	Target
ORAND	3	3	2	92	100	50	binary
ANDOR	4	4	2	90	100	50	binary
ADDER	3	3	2	92	100	50	4-class
LED-16	16	16	2	66	100	180	36-class
KDD	na	na	na	na	39	145,586	23-class

The ORAND dataset contains three relevant features $X_{1},X_{2}$, and $X_{3}$. The target variable *Y* is calculated via the following formula:

$$Y=X_{1}\wedge (X_{2}\vee X_{3}).$$

The ANDOR dataset contains four relevant features $X_{1},X_{2},X_{3}$, and $X_{4}$. The target variable *Y* is calculated via the following formula:

$$Y=(X_{1}\wedge X_{2})\vee (X_{3}\wedge X_{4}).$$

The ADDER dataset has three inputs $X_{1},X_{2}$, and $X_{3}$ and produces two outputs $Y_{1}$ and $Y_{2}$. The outputs are calculated according to the following formulae:

$$Y_{1}=X_{1}⊕X_{2}⊕X_{3}\;Y_{2}=(X_{1}\wedge X_{2})\vee (X_{3}\wedge (X_{1}⊕X_{2})).$$

By combining the values of $Y_{1}$ and $Y_{2}$ into a single target variable $Y=(Y_{1},Y_{2})$, we obtain a 4-class target variable: Y={(0,0),(0,1),(1,0),(1,1)}.

The LED-16 dataset is based on the 16-segment display configuration (Fig. 6). which allows the display of all 26 letters of the English alphabet as well as all the digits 0-9. Each segment represents a binary feature: on/off. The target variable is the alpha-numeric value displayed by the segments. The complete details of the synthetic datasets including the source code are available in [27], [28].

### 4.2 Benchmarking

We benchmark NES against several popular feature selection algorithms: maximum relevance minimum redundancy algorithm (mRMR), Boruta, genetic feature selection, Lasso, Elastic Net, and recursive feature elimination (RFE). mRMR is a highly recognized algorithm that is used both in industry and academia [8], [49]. It is a sequential forward feature selection algorithm. At each iteration, the feature with the highest relevance with respect to the target variable and the lowest redundancy with respect to the already chosen subset of features is selected. The relevance and redundancy can be computed using different metrics though mutual information is the most frequently used approach for categorical data. The implementation of the mRMR algorithm used in our study is obtained from [32].

The Boruta algorithm is a well-known wrapper method that compares the importance of the original features to the importance of shadow features [30], [44]. The shadow features are defined as randomized copies of the original features. Features that have higher importance than the highest ranked shadow feature are selected. The selection process is repeated several times. The final determination of statistically significant features is made based on the binomial distribution of the hits. The implementation of the Boruta algorithm used in our study is obtained from [20].

Genetic algorithms represent stochastic optimization framework inspired by the processes in evolutionary biology and have gained a large attention in recent years [29], [42], [45]. Genetic feature selection algorithms often consist of 5 main stages - initialization, fitness assignment, selection, cross-over, and mutation. The last 4 stages are repeated several times to mimic evolutionary cycles. The appropriate implementation of the genetic algorithm using cross-validation is obtained from [12].

Recursive Feature Selection (RFE) is a popular technique for feature selection that iteratively ranks and selects the most important features. It starts with the set of all features and uses a chosen estimator to evaluate feature importance. The estimator is often chosen to be Support Vector Machines and feature importance is measured by the coefficient value in the estimated model [19]. The least important features are removed, and the model is retrained. The process continues until a specified number of features is selected.

Lasso is a well known regularization technique that is used in various machine learning models. In classification, it can be used to select relevant features and reduce model complexity by adding a penalty term to the logistic regression cost function [17]. The penalty term is the $L_{1}$ norm (sum of absolute values) of the regression coefficients multiplied by a regularization parameter *λ*. The $L_{1}$ norm encourages sparsity in the coefficient vector, which means it pushes the coefficients of irrelevant features towards zero, effectively performing feature selection.

Elastic Net is another commonly used regularization technique for feature selection [7]. It combines both $L_{1}$ (Lasso) and $L_{2}$ (Ridge) regularization penalties to address the limitations of each method and provide a more flexible approach to feature selection. It can handle situations where there are correlated features or when the number of features is larger than the number of samples.

### 4.3 Experimental setup

In the numerical experiments, the proposed NES algorithm is compared to the benchmark methods mRMR, Boruta, Genetic, RFE, Lasso, and Elastic Net. The benchmark methods are employed using mostly their default settings as per the original source. The details of the benchmark methods are provided in Table 3. The code for the numerical experiments is publicly available on GitHub [34], where more details regarding the settings are provided.

**Table 3:** The source and the settings of the benchmark algorithms.

Algorithm	Source	Settings
NES	Section 3[34]	
mRMR	Mazzanti (2022) [32]	default
Boruta	Homola (2022) [20]	default
Genetic	Calzolari (2022) [12]	default
RFE	Scikit [36]	LinearSVC, default
Lasso	Scikit [36]	LinearSVC, C=0.1, default
ElasticNet	Scikit [36]	LogisticRegression, C=0.2, l1_ratio=0.5

Each method is applied to the datasets in Table 2 and the optimal feature subsets are selected. In the case of the synthetic datasets, where the relevant features are known, the precision and recall are calculated directly based on the selected feature subsets. Precision is calculated as follows:

$$\text{Precision}=\frac{\mathrm{Relevant}\;\;\mathrm{Selected}}{\mathrm{Total}\;\;\mathrm{Selected}},$$

where *Relevant Selected* is the number of relevant features selected and *Total Selected* is the total number of features in the dataset. Note that the datasets contain a redundant copy of each relevant feature, so it is acceptable for an algorithm to select a redundant feature instead of the corresponding relevant feature. However, in case both the relevant feature and its redundant copy are selected, only the relevant is counted. Recall is calculated as follows:

$$\text{Recall}=\frac{\mathrm{Relevant}\;\;\mathrm{Selected}}{\mathrm{Total}\;\;\mathrm{Relevant}},$$

where *Total Relevant* is the total number for relevant features in the dataset.

The precision and recall score can be used to calculate the balanced F1-score given by the following equation:

$$\text{F1-score}=2\cdot \frac{\text{Precision}\cdot \text{Recall}}{\text{Precision}+\text{Recall}}.$$

In case of KDD, the data is first split into train and test subsets. The train set is used to select the relevant features. Then, random forest classifier is trained using the selected features in the train set. Finally, the trained classifier is evaluated on the test set using the selected features. The precision, recall, and F1-score metrics are calculated based on the results of the test set.

### 4.4 Results

#### 4.4.1 ORAND

**Figure 2:**
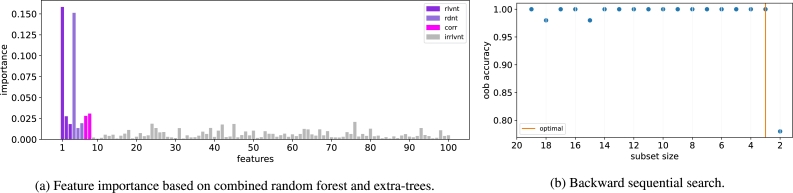
The two-stage NES algorithm for ORAND dataset.

We begin our discussion with results using the ORAND dataset. In applying the NES algorithm, first, we calculate the individual feature importances using random forest and extra-trees classifiers based on Equation (1). As shown in Fig. 2a, while the individual feature scores are able to distinguish between the relevant and the majority of the irrelevant variables, they fail to separate the relevant variables from redundant and correlated features. Thus, in the second stage of the selection process, we perform backward sequential feature selection using the top 20 features that are selected based on the individual scores in Fig. 2a. In particular, at each iteration, the least important feature - determined based on the out-of-bag accuracy of random tree classifier - is removed from the feature subset.

As shown in Fig. 2b, there is a sharp drop in accuracy between subset size 3 and 2. It indicates that 3 is the optimal size of the feature subset. Indeed, the ORAND dataset contains 3 relevant and 3 redundant variables (Table 2). Thus, the proposed approach is able to detect the correct size of the optimal subset. It remains to validate that NES selects the correct features in the optimal subset. To be sure, the proposed method selects two relevant variables 1 and 2 together with the redundant variable 6. However, variable 6 corresponds to the relevant variable 3. Therefore, in fact, the NES algorithm correctly selects all three relevant variables in the dataset.

The comparison of the features selected by NES and those selected by the benchmark algorithms mRMR, Boruta, Genetic, RFE, Lasso, and Elastic Net is presented in Table 4. Note that both mRMR and RFE provide feature rankings. For instance, feature 3 is ranked second by mRMR. On the other hand, Boruta, Genetic, Lasso, and Elastic Net provide selected features in no particular order. As shown in Table 4, while mRMR, correctly ranks the relevant features at the top, it also assigns high ranking to several irrelevant, correlated, and redundant features. Similarly, Lasso and Elastic Net correctly identify the relevant features but also select irrelevant, correlated, and redundant features. The Boruta algorithm selects a limited number of features, but only one of the selected features is relevant. Similarly, the Genetic algorithm is able to identify only one of the relevant variables.

**Table 4:** Comparison of the selected features by NES and the benchmark algorithms on the ORAND dataset.

Algorithm	Selected features
Relevant	1, 2, 3
NES	1, 2, 3
mRMR	1, 3, 2, 4, 42, 7, 8, 39, 94, 31
Boruta	1, 4, 7, 8
Genetic	4, 13, 24, 87
RFE	2, 15, 11, 1, 35, 4, 9, 29, 95, 54
Lasso	1, 2, 3, 4, 5, 6, 8, 24
ElasticNet	1, 2, 3, 4, 5, 6, 7, 8, 24, 42

#### 4.4.2 ANDOR

**Figure 3:**
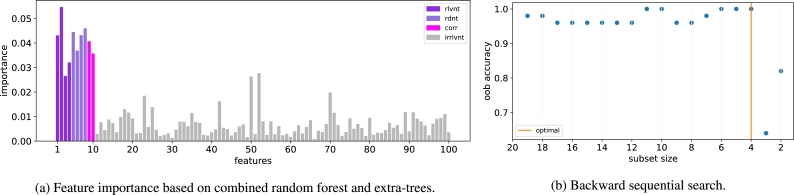
The two-stage NES algorithm for ANDOR dataset.

The feature scores for the ANDOR dataset are presented in Fig. 3a. It can be seen that the relevant features tend to score higher than the irrelevant features. However, the redundant and correlated feature scores are as high as the relevant features. Therefore, we select the top 20 features for downstream analysis.

The accuracy of subsets in the backward sequential search is presented in Fig. 3b. There is a sharp drop in accuracy between subset size 4 and 3. It indicates that the optimal size of feature subset is 4. Since there are in fact 4 relevant features in the ANDOR dataset, our approach correctly identifies the size of the optimal subset. Furthermore, the selected subset contains features 1, 2, 4 and 7. The features 1, 2, and 4 are relevant. Feature 7 is a redundant feature which corresponds to the relevant feature 3. Thus, the proposed approach selects the correct features.

The comparison of the features selected by NES and those selected by the benchmark algorithms is presented in Table 5. We observe that none of the benchmark algorithms provide a prefect selection. While the mRMR and Boruta algorithms do select the relevant features, they also assign high ranking to several irrelevant, correlated, and redundant features. Similarly, Lasso and Elastic Net select the relevant but also several extra features. While RFE ranks the relevant features in the top 10, it fails to assign them the highest level of importance. The Genetic algorithm selects a small group of features and correctly identifies two relevant variables.

**Table 5:** Comparison of the selected features on the ANDOR dataset.

Algorithm	Selected features
Relevant	1, 2, 3, 4
NES	1, 2, 3, 4
mRMR	9, 3, 1, 2, 50, 4, 52, 5, 10, 6
Boruta	1, 2, 3, 4, 5, 6, 7, 8, 9, 10
Genetic	3, 4, 50
RFE	9, 8, 20, 10, 5, 4, 2, 3, 7, 15
Lasso	1, 2, 3, 5, 6, 7, 8, 9, 10, 42, 50, 52
ElasticNet	1, 2, 3, 4, 5, 6, 7, 8, 9, 10, 42, 50, 52

#### 4.4.3 ADDER

**Figure 4:**
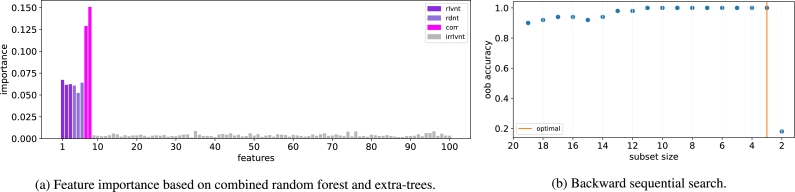
The two-stage NES algorithm for ADDER dataset.

The feature scores for the ADDER dataset are presented in Fig. 4a, where it can be seen that there is a significant difference between the relevant and irrelevant features. On the other hand, there is no difference between the relevant and redundant features. Moreover, the correlated features attain the highest scores. Therefore, a second stage of selection is required to separate the relevant variables from the redundant and correlated variables.

The second stage of feature selection is illustrated in Fig. 4b, where we observe a sharp drop in accuracy between subset size 3 and 2 which implies that the optimal size of feature subset is 3. Indeed, the ADDER dataset contains 3 relevant variables. Furthermore, the optimal chosen subset of size 3 consists of the redundant features 4, 5, and 6 which correspond to the relevant features 1, 2, and 3. Thus, the proposed NES algorithm selects all the correct features.

The comparison of the features selected by NES and those selected by the benchmark algorithms is presented in Table 6. As in the previous datasets, none of the benchmark algorithms provide a prefect selection. In particular, the mRMR algorithm ranks several irrelevant features ahead of the relevant variables, while Boruta selects all the redundant and correlated features together with the relevant variables. The Genetic algorithm selects a small group of features, but is able to identify only one relevant feature. RFE performs relatively well by ranking the relevant features at the top. Similarly, Elastic Net correctly identifies all the relevant features albeit with a few extra variables.

**Table 6:** Comparison of the selected features on the ADDER dataset.

Algorithm	Selected features
Relevant	1, 2, 3
NES	1, 2, 3
mRMR	7, 94, 77, 8, 31, 67, 1, 3, 2, 4
Boruta	1, 2, 3, 4, 5, 6, 7, 8
Genetic	4, 8, 17, 34, 78
RFE	6, 5, 4, 3, 1, 2, 14, 8, 86, 47
Lasso	1, 7, 8, 59, 77, 88, 98
ElasticNet	1, 4, 6, 7, 8, 85, 98

#### 4.4.4 LED-16

**Figure 5:**
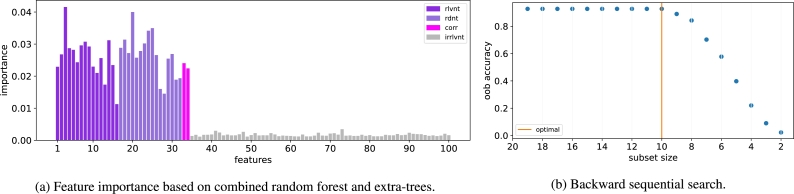
The two-stage NES algorithm for LED-16 dataset.

The LED-16 dataset is significantly different from the previous datasets. In particular, it is a multi-class dataset with 32 different values of the target variable. It has 16 original features. The relevant features have different levels of relevance in that they affect different number of target values. The feature scores for the LED-16 dataset are presented in Fig. 5a, where it can be seen that the relevant features have significantly higher scores than the irrelevant features. However, the redundant and correlated features have high scores that are similar to the relevant features.

The results of the backward sequential feature selection are presented in Fig. 5b, where we observe a decrease in accuracy beginning at subset size 10 which indicates the optimal subset size. In fact, there are 14 relevant variables, so our approach underestimated the true size of the optimal subset. On the other hand, all the features selected in the optimal subset of size 10 are relevant, i.e., no redundant, correlated, or irrelevant features are selected in the final subset. Finally, test results show that the accuracy of the selected subset (0.9417) is equal to the accuracy of the full feature set (0.9417). So while NES did not identify all the relevant variables, it selected the most relevant features.

**Figure 6:**
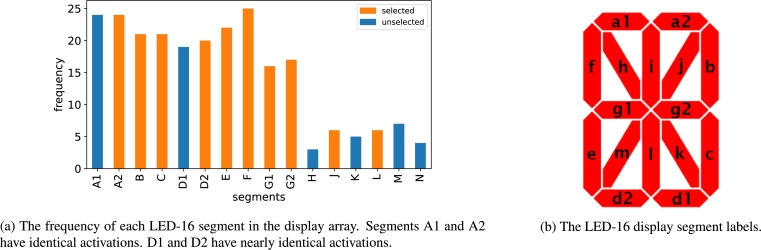
The LED-16 display segments.

The features selected in the optimal subset are presented in Fig. 6. It can be seen that all the high frequency display segments are selected by the NES algorithm. Note that A1 and A2 have identical activations, i.e., they light up simultaneously. Segments D1 and D2 also activate simultaneously except for a single character display. Thus, A1 and D1 are practically redundant features.

The comparison of the features selected by NES and those selected by the benchmark algorithms is presented in Table 7. The mRMR and Boruta algorithms rank all first 32 features equally at the top. In other words, while the algorithms separate the relevant features from the irrelevant features, they fail to distinguish the relevant features from the redundant and correlated features. The Genetic algorithm achieves high precision by selecting only 1 irrelevant variable, but at the same time it fails to identify 5 relevant features. Similarly, RFE performs well by achieving perfect precision. However, it is not able to capture 4 relevant variables. Lasso and Elastic Net achieve mixed results with moderate precision and recall. The results of the LED-16 dataset demonstrate the advantage of the NES algorithm in multi-class settings.

**Table 7:** Comparison of the selected features on the LED-16 dataset.

Algorithm	Selected features
Relevant	2, 3, 4, 6-16
NES	2, 3, 4, 6-10, 12, 14
mRMR	1-32
Boruta	1-34
Genetic	2, 3, 6, 7, 9, 15, 20, 24, 26, 83
RFE	24, 20, 11, 14, 9, 17, 12, 19, 13, 18
Lasso	9, 10, 19, 21, 23, 24, 33, 34, 35, 55
ElasticNet	3, 5, 6, 7, 8, 9, 10, 23, 25, 26, 33, 34, 73

#### 4.4.5 KDD

The final dataset used in our study is the well-known KDD dataset which simulates network intrusions in a military environment [43]. There are 39 features and 23 target classes. It is a highly imbalanced dataset that is heavily dominated by *normal* and *neptune* classes. Class membership ranges from 2 (*spy*) to 87,832 (*normal*) instances. The dataset is split into train and test subsets (70/30). The train set is used to select the important features, while the test set is used to evaluate the accuracy of the trained classifier based on the selected features.

**Figure 7:**
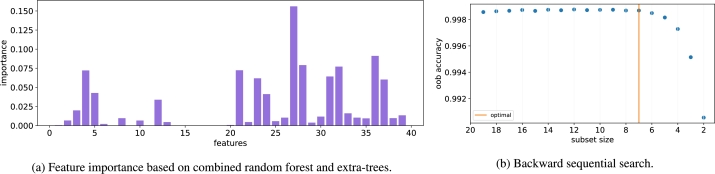
The two-stage NES algorithm for KDD dataset.

The feature scores for the KDD dataset are presented in Fig. 7a. It can be seen that while some features have high scores (27, 28, 31, 32, 36, 37), other feature scores are nearly zero (1, 7, 9, 11, 14-20). The results of the backward sequential feature selection are presented in Fig. 7b, where we observe a decrease in accuracy beginning at subset size 7 which indicates the optimal subset size. The features selected in the optimal subset are: service, src_bytes, dst_host_count, dst_host_diff_srv_rate, dst_host_same_src_port_rate, dst_host_serror_rate, dst_host_rerror_rate.

Comparison of the features selected by NES and those selected by the benchmark algorithms together with the corresponding accuracy on the test set is presented in Table 8. The results show that the accuracy of the NES-selected subset (0.9983) is close to that of the full feature set (0.9989). For comparison, the accuracy of the top 10 features selected by mRMR is lower by 1.4% (0.9844). Similarly, the Genetic algorithm achieves lower accuracy than NES by 1.5%. While the Boruta algorithm achieves the same accuracy as NES, it selects almost the entire set of features which is not practical. Similarly, Lasso and Elastic Net achieve nearly the same accuracy as NES but using a large number of features. The results show that NES performs well on the KDD dataset. The 7 features selected by the NES algorithm achieve almost the same accuracy as the full feature set.

**Table 8:** Comparison of the accuracy of the selected features.

Algorithm	Number of selected features	Accuracy
Full set	39	0.9989
NES	7	0.9983
mRMR	10	0.9844
Boruta	34	0.9983
Genetic	9	0.9834
Lasso	23	0.9985
ElasticNet	23	0.9985

### 4.5 Discussion

To evaluate the proposed NES algorithm, we conducted numerical experiments on 5 datasets. The proposed algorithm was benchmarked against several popular existing algorithms: mRMR, Boruta, Genetic, RFE, Lasso, and Elastic Net. The precision and recall of the features selection algorithms are presented in Table 9, Table 10. The precision is calculated as the number of relevant variables selected divided by the total number of variables selected. The recall is calculated as the number of relevant variables selected divided by the total number of relevant variables.

**Table 9:** The precision on the four synthetic datasets and accuracy on the KDD dataset of the features selection methods.

Dataset	NES	mRMR	Boruta	Genetic	RFE	Lasso	ElasticNet
ORAND	1.0	0.3	0.25	0.25	0.20	0.38	0.30
ANDOR	1.0	0.4	0.4	0.67	0.40	0.33	0.31
ADDER	1.0	0.3	0.375	0.2	0.30	0.14	0.28
LED-16	1.0	0.44	0.41	0.6	0.90	0.50	0.46
KDD*	0.9983	0.9844	0.9983	0.9834

**Table 10:** The recall levels of the feature selection algorithms.

Dataset	NES	mRMR	Boruta	Genetic	RFE	Lasso	ElasticNet
ORAND	1.0	1.0	0.33	0.33.	0.67	1.0	1.0
ANDOR	1.0	1.0	1.0	0.5	1.0	1.0	1.0
ADDER	1.0	1.0	1.0	0.33.	1.0	0.33	0.67
LED-16	0.71	1.0	1.0	0.43	0.64	0.36	0.43

As shown in Table 9, NES achieves perfect precision on all four synthetic datasets. In other words, NES does not select any extraneous features. It is a remarkable result given that the datasets also contain redundant and correlated features. Similarly, the proposed method achieves perfect recall on the ORAND, ANDOR, and ADDER datasets. In other words, NES is able to identify all the relevant features in the data. The results indicate that the proposed approach is particularly well suited for low dimensional target variables.

In the LED-16 dataset, NES selects 10 of the 14 relevant features (recall 0.71). On the other hand, the feature subset selected by the algorithm has the same test accuracy (0.9417) as the full features set (0.9417). So while NES does not capture all the relevant features it still achieves the same classification accuracy as the full feature set. Note that the algorithm did not select any extraneous variables (precision 1). As mentioned in the previous section, LED-16 dataset contains features of varying importance (Fig. 6a) so it is likely that the algorithm could not identify the feature with low importance.

In the last experiment, we employed the KDD dataset where the relevant features are unknown. Since the nature of the features is unknown, we rely on the test accuracy to evaluate the selected feature subset. As shown in Table 9, NES selected 7 features that produce test accuracy that is very close to that of the full feature set. The results show that the algorithm selected the optimal, or near optimal, subset of features.

Comparison of NES against the benchmark algorithms in Table 9 reveals that the greatest advantage of the proposed method is its precision. In particular, mRMR, Boruta, Lasso, and Elastic Net do not exceed precision level of 0.50 on any of the tested datasets. In other words, more than half of the selected features are extraneous. Similarly, the precision of the Genetic algorithm ranges between 0.2 and 0.67. RFE is able to achieve relatively high precision albeit only on a single dataset. On the other hand, NES achieves perfect precision all four synthetic datasets. As shown in Table 10, the recall levels of NES, mRMR, and Boruta are near perfect, while the Genetic algorithm fails to recall more than half of the relevant features. The RFE, Lasso, and Elastic Net algorithms attain mixed recall levels.

The results of F1-score presented in Table 11 demonstrate the effectiveness of the NES algorithm. The proposed method achieves the highest F1-score on every dataset. Since F1-score is based on the combined values of precision and recall, it reflects the overall performance of the algorithms. We conclude that NES significantly outperforms all the benchmark methods.

**Table 11:** The F1-score of the feature selection algorithms.

Dataset	NES	mRMR	Boruta	Genetic	RFE	Lasso	ElasticNet
ORAND	1.0	0.30	0.08	0.08	0.13	0.38	0.30
ANDOR	1.0	0.40	0.40	0.34	0.40	0.33	0.31
ADDER	1.0	0.30	0.38	0.07	0.30	0.04	0.19
LED	0.71	0.44	0.41	0.26	0.58	0.18	0.20

It is important that a distinction is made between the existing and the proposed method. To be sure, there are significant differences between the proposed NES method and other current feature selection techniques. The distinction is two-fold. First, the proposed approach is algorithmically new in its simplicity and the use of double feature importance scoring which reduces the variance of the results. Unlike other ensemble methods that employ multi-stage processes to execute the algorithm, the NES algorithm proposes an uncomplicated, two-stage algorithm that is easy to deploy. Furthermore, the balanced scoring system based on the reduction in impurity using the random forest and extra-trees classifiers provides a stable scoring mechanism. Second, the proposed algorithm is different in its superior performance. The empirical tests (Table 9, Table 10, Table 11) demonstrate that the NES algorithm is significantly more accurate than the popular benchmark algorithms. It is particularly effective in discarding the redundant and correlated features.

## 5 Conclusion

In this paper, we proposed a novel ensemble-based feature selection algorithm called Nested Ensemble Selection (NES). The NES algorithm differs from the existing filter-wrapper methods in its simplicity and efficiency as well as near-perfect accuracy. Extensive empirical testing against several popular benchmark algorithms demonstrated the superiority of the proposed method, especially in multi-class datasets.

The proposed method was tested on 5 different datasets. The results showed that NES is capable of achieving outstanding and desirable results both in identifying the relevant features as well as classification accuracy (Table 9, Table 10). For comparison, NES was benchmarked against mRMR, Boruta, Genetic, RFE, Lasso, and Elastic Net algorithms and demonstrated vastly superior precision. In addition, NES provides a robust mechanism for determining the size of the optimal feature subset based on the graph of the test accuracy.

As a future research avenue, extending NES to regression problems can be explored. Since the main components of NES, namely random forest and extra-trees classifiers, do exist for regression tasks it would be logical to research this in the future. The filter-wrapper technique can be used in other application where the complexity of the problem can be reduced.

## CRediT authorship contribution statement

**Firuz Kamalov:** Conceived and designed the experiments; Wrote the paper; Analyzed and interpreted the data. **Hana Sulieman**, **Sherif Moussa**, **Murodbek Safaraliev:** Contributed reagents, materials, analysis tools or data; Wrote the paper. **Jorge Avante Reyes:** Performed the experiments; Wrote the paper.

## Declaration of Competing Interest

The authors declare that they have no known competing financial interests or personal relationships that could have appeared to influence the work reported in this paper.

## Data Availability

Data associated with this study has been deposited at https://github.com/group-automorphism/synthetic_data.
